# Differentiating anxiety from fear: an experimental–pharmacological approach

**DOI:** 10.1017/pen.2020.1

**Published:** 2020-06-17

**Authors:** Julia V. Lippold, Ulrich Ettinger, René Hurlemann, Philip J. Corr, Martin Reuter, Adam M. Perkins

**Affiliations:** 1 Department of Psychology, University of Bonn, Bonn, Germany; 2 Department of Psychiatry and Psychotherapy, University of Bonn, Bonn, Germany; 3 Department of Psychiatry, University of Oldenburg, Oldenburg, Germany; 4 Department of Psychiatry, University of Oldenburg Medical Campus, Bad Zwischenahn, Germany; 5 Division of Medical Psychology, Department of Psychiatry, University Hospital Bonn, Bonn, Germany; 6 Department of Psychology, City, University of London, London, UK; 7 Center for Economics and Neuroscience, University of Bonn, Bonn, Germany; 8 Department of Psychological Medicine, Institute of Psychiatry, King’s College London, London, UK

**Keywords:** Fear, Anxiety, Revised-reinforcement-sensitivity-theory, Lorazepam

## Abstract

Gray’s theory of personality postulates that fear and anxiety are distinct emotional systems with only the latter being sensitive to anxiolytic drugs. His work was mainly based on animal research, and translational studies validating his theory are scarce. Previous work in humans showed an influence of the benzodiazepine lorazepam on both systems, however, dependent on dosage (1 and 2 mg) and personality differences in negative emotionality. The present study aims to replicate these findings, and in addition tests the drug threshold effect by introducing a lower dosage of 0.5 mg lorazepam. Fifty healthy adults (23 males, age_mean_ 22.40, SD ± 3.68) participated in an experimental threat-avoidance paradigm designed to dissociate risk assessment intensity (RAI, reflecting anxiety) and flight intensity (FI, reflecting fear) and completed two self-report questionnaires assessing facets of negative emotionality (*Spielberger State Trait Anxiety Inventory* and *Fear Survey Schedule*). In a randomized placebo-controlled within-subjects design, 0.5 and 1 mg of lorazepam were applied per os. Saccadic peak velocity was assessed by means of eye-tracking in order to control for sedating drug effects. Results showed the expected and specific anxiolytic effect of lorazepam on RAI, however, only in the 0.5 mg condition. FI was not influenced by lorazepam, and previous findings of interaction effects of lorazepam with self-reported negative emotionality could not be corroborated. Overall, this study demonstrates anxiolytic effects of lorazepam in dosages ≤1 mg in the absence of a drug effect on fear using a translational behavioural task. However, a putative moderating role of personality on defensive behaviour has to be clarified in future research.

## 1. Theoretical Background

The revised reinforcement sensitivity theory (r-RST) postulates that fear and anxiety are two distinct neural affective systems (Gray & McNaughton, 2000). This hypothesis was based on extensive animal research using pharmacological approaches. Anxiety is thought to be related to defensive approach behaviour (comprising passive avoidance behaviour), whereas fear is hypothesized to be related to defensive active avoidance behaviour. Stimuli that are new or ambiguous, that is, where the potential consequences are unclear, stimulate the behavioural inhibition system (BIS; the r-RST’s proxy for anxiety) and lead to increased attention, risk assessment and checking of memory traces to investigate the potential threat of the stimuli. The BIS is also activated by all kinds of goal conflicts, including approach–avoidance, avoidance–avoidance and approach–approach conflicts. These conflicts represent uncertainty and are accompanied by feelings of anxiety. In contrast, the fear system is triggered by clear negative stimuli that need not be approached, but simply have to be avoided, or escaped from. Defensive avoidance comprises three potential defensive behavioural consequences: fight, flight and freeze (FFF-system; the r-RST’s proxy for fear). The perceived distance from the threat crucially influences the choice of the respective behavioural alternatives. Freezing behaviour is activated if the perceived threat is close and impossible to escape. At a greater perceived distance of the threat and if there is a possibility to escape, flight behaviour is elicited. However, when the threat is so close that flight is not possible and freezing is not an appropriate reaction, defensive threat occurs (such as vocalization and display of teeth or claws), followed by explosive defensive attack at zero distance to threat (i.e. when nose-to-nose), that is, fight behaviour.

The hypothesis that fear and anxiety are distinct emotional systems is supported by experimental pharmacological work, demonstrating that only anxiety but not fear can be influenced by anxiolytic drugs (Gray & McNaughton, 2000).

Gray’s r-RST was strongly influenced by seminal work by Blanchard and Blanchard (1989), which showed that anxiety and fear behaviour could be successfully differentiated in rodents. Blanchard et al. (1990a) investigated anxiolytic drug effects with respect to risk assessment behaviour in rodents. Rodents were exposed to different types of threats reaching from mild threat (e.g. the odour of a predator) to severe threat (e.g. clear sights of a nearby predator) representing anxiety and fear, respectively. The results showed that the rodents’ state level of defensiveness determined the direction of anxiolytic drug effects: The risk assessment of mildly threatened rodents was reduced (Blanchard, Blanchard, Weiss & Meyer, 1990a), while the risk assessment of severely threatened rodents was increased by anxiolytics (Blanchard, Blanchard, Tom & Rodgers, 1990b).

Despite convincing rodent studies, approaches attempting to extrapolate these findings to humans are scarcer (e.g. Reuter, Schmitz, Corr & Hennig, 2006). One of the most severe problems of such translational work is to transform the phenotypes under investigation from rodents to humans and vice versa. However, recent neuroimaging studies have begun to test the neural networks Gray postulates for his distinct affective systems (Mobbs et al., 2010; Montag, Basten, Stelzel, Fiebach & Reuter, 2010; Patrick et al., 2019; Reuter et al., 2004) and there are also approaches that tested the sensitivity to pharmacological challenges. For example, Grillon et al. (2006) investigated the effects of the benzodiazepine alprazolam on cued fear and contextual anxiety in a startle reflex paradigm containing three different conditions. The conditions included predictable electric shock (i.e. fear), unpredictable electric shock (i.e. anxiety) and no electric shock. Results confirmed the assumption that cued fear is not sensitive to alprazolam, whereas contextual anxiety is influenced by alprazolam. Moberg and Curtin (2009) examined the effect of alcohol on startle responses in experimental conditions with predictable and unpredictable administration of electric shock. The authors demonstrated that alcohol reduced the startle response in unpredictable conditions but not in predictable conditions.

To further extent these translational findings in humans, we adopted and translated a rodent model of fear and anxiety, the *Mouse Defense Test Battery* (Blanchard, Griebel & Blanchard, 2003) into the *Joystick Operated Runway Task* (JORT; Perkins et al., 2009). The JORT paradigm attempts to operationalize the measurement of dissociable components of fear (flight intensity, FI) and anxiety (risk assessment intensity, RAI) in humans [for a detailed description (Methods section) and a critical review (Discussion section) of this task see below].

In a first placebo-controlled pharmacological study on the JORT, the effects of citalopram (10 mg) and lorazepam (1 mg) were investigated in 30 healthy males. We hypothesized that lorazepam, a benzodiazepine drug with well-established anxiolytic properties, would alter behaviour reflecting anxiety but not fear. Conversely, we hypothesized that citalopram, a selective serotonin reuptake inhibitor with well-established anti-panic properties, would alter fear behaviour but not anxiety behaviour. To test the interaction between severity of stress and lorazepam, we considered individual differences in human trait perception of threat intensity. To this we administered the *Fear Survey Schedule* (FSS; Wolpe & Lang, 1974), which consists of two major subscales: First, social fear measures a mixture between trait anxiety and fear (Cooper, Perkins & Corr, 2007); and second, tissue-damage fear which constitutes a relatively pure measure of fear (Perkins, Kemp & Corr, 2007). Furthermore, we included the Y2 (trait) scale from the *Spielberger State-Trait Anxiety Inventory* (STAI, Spielberger, Gorsuch, Lushene, Vagg & Jacobs, 1983) in order to have a general measure of anxiety. We found support that lorazepam modulated RAI. In addition, a particular strong anxiety reducing effect was observable in participants scoring in the lower half of the sample on FSS social fear. These findings were in line with prior delineated assumptions and provided further evidence that human (trait) personality differences are comparable to experimentally induced (state) anxiety differences in rodents. Contrary to our prior assumption, citalopram showed an effect neither on RAI nor on FI. For this reason, we did not test citalopram in follow-up studies.

In 2013, we built on our previous findings and tested the effects of two different doses of lorazepam (1 and 2 mg) versus placebo (Perkins et al., 2013) in 40 healthy adults (20 females). Here, we assumed that questionnaire scores of STAI-T would correlate positively with RAI, whereas questionnaire scores of FSS would correlate positively with FI. We additionally administered the Eysenck Personality Questionnaire – Revised (Eysenck & Eysenck, 1991) to include neuroticism as a superordinate measure of proneness to negative emotionality. We built our hypotheses on the assumption of 2009. Therefore, lorazepam should increase RAI in participants scoring high on trait anxiety but decrease RAI in participants scoring low on trait anxiety. Furthermore, in line with Gray’s theory, we did not expect an anxiolytic effect of lorazepam on FI. As hypothesized, RAI was affected by lorazepam but the effect was modulated by personality. A dose of 2 mg lorazepam reduced RAI in low scorers on trait anxiety but increased RAI in high scorers, whereas personality had no differential effects in the placebo and 1 mg lorazepam conditions. Against our predictions, there was an interaction effect between lorazepam condition and tissue-damage fear. Lorazepam increased FI in the participants with low scores on FSS tissue damage in a dose-dependent manner, whereas FI was decreased in participants with high FSS tissue damage scores in a dose-dependent manner.

In sum, two consecutive pharmacological studies showed an effect of lorazepam on defensive behaviour as measured by the JORT, but the patterning of those effects varied between the two studies. In the first study, 1 mg of lorazepam reduced behavioural anxiety (RAI) but only in participants scoring low on the FSS social fear, a hybrid measure of anxiety and fear. Conversely, in the second study, only the dosage of 2 mg of lorazepam reduced RAI, but this time only in participants scoring low in STAI-T. Furthermore, in Study 2, against our theoretical predictions, a behavioural measure of fear (FI) was influenced dependent on dosage of lorazepam and scores of the FSS tissue-damage fear scale. FI showed a dose-dependent increase in participants scoring low and a dose-dependent decrease in high-scoring participants.

Therefore, although both studies showed some overlap with respect to their results, there are also some discrepancies especially regarding which dosage of lorazepam showed significant effects on defensive behaviour.

The present study was designed to further elucidate the effects of lorazepam on defensive behaviour and its interaction with personality in an independent study with an increased sample size. We aimed to balance gender and introduced a lower dosage of 0.5 mg lorazepam in order to obtain information on lorazepam’s low range threshold effects by comparing it to the 1 mg and the placebo condition.

## 2. Materials and Methods

### 2.1 Recruitment, inclusion and exclusion criteria

Participants were recruited through social media (e.g. Facebook), student newsletters of the University of Bonn and flyers. For inclusion, participants had to be between 18 years and 35 years old, healthy and right-handed. Exclusion criteria were consumption of any medication (except of oral contraceptives in women), a current or past diagnosis of physical, neurological or psychiatric condition, blood pressure below 100/60 or above 130/90, a body mass index of less than 18 or more than 30, injuries or diseases of the inner ear accompanied by loss of hearing, colour-blindness, current or recent (within the last 12 months) consumption of drugs or nicotine, earlier consumption of lorazepam or other benzodiazepines during lifetime and for women a positive pregnancy test. The study was approved by the research ethics committee of the Faculty of Medicine at the University of Bonn.

The final sample consisted of 50 healthy participants (23 males; mean age 22.40 years, SD ± 3.68). All were students of the University of Bonn or neighbouring universities.

### 2.2 Screening

First, participants completed an online questionnaire intended to verify whether they met the inclusion criteria and none of the exclusion criteria. If so, participants were invited to a screening in person to further ensure that they fulfil all conditions of participation. They first gave written, informed consent and declared their willingness in principle to participate in the experiments. Then, they were screened with in-depth interviews for the existence of any psychiatric, neurological or physical disorders. Height, weight and blood pressure were measured. The absence of colour-blindness was verified by two red–green pictures of the Ishihara colour test. Additionally, the absence of drug consumption and, in females, the absence of pregnancy were tested. Finally, all participants completed the Edinburgh Handedness Inventory (Oldfield, 1971), the FSS and the STAI-T.

### 2.3 Study protocol

We used a double-blind, placebo-controlled, randomized, within-subjects design. Each participant received placebo, 0.5 and 1 mg lorazepam, with order of administration following a Latin square design. Assessments were scheduled a week apart to allow drug washout.

After arriving in the laboratory, participants were once again briefly checked for health. Female participants underwent urine pregnancy tests before all three experimental sessions to confirm the absence of pregnancy. During the assessments, participants were allowed to drink as much still water as they liked but were not allowed to eat or drink anything else. Tablets were administered with still water and, due to our double-blind study design, were contained in opaque capsules. Due to the pharmacokinetics of lorazepam, there was a 2 h wait from capsule administration until the actual experimental session started (Kyriakopoulos, Greenblatt & Shader, 1978).

**Figure 1.**
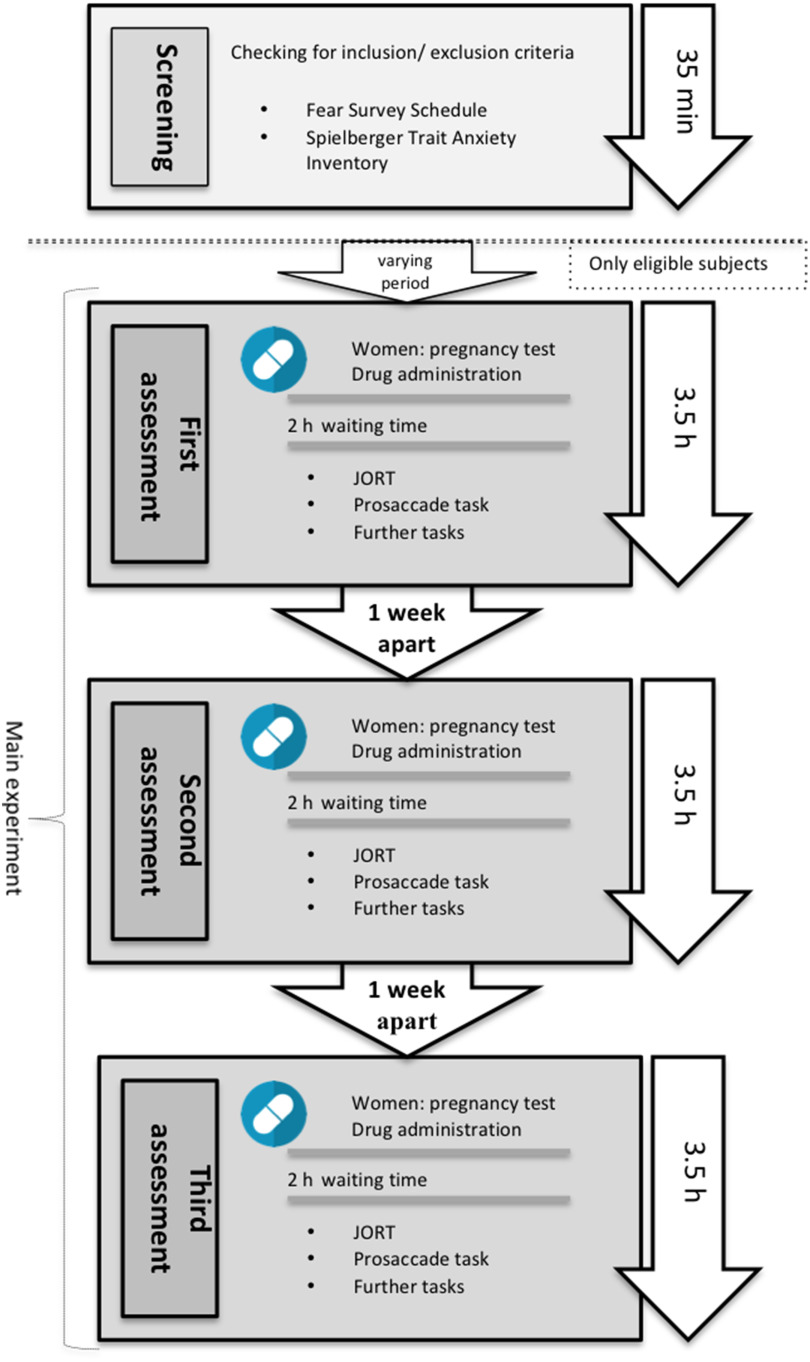
Study protocol. Further tasks: saccadic adaptation task, antisaccade task, Simon task, flanker task.

All assessments started with JORT, followed by the saccade task. JORT took approximately 20 min and saccades a further 2 min. Subsequently, participants carried out four further tasks not reported in this paper (saccadic adaptation task, antisaccade task, Simon task, flanker task). An overview on the study protocol can be seen in Figure 1.

### 2.4 Joystick Operated Runway Task

Human defensive behaviour was measured by the JORT (Perkins et al., 2009, 2013). The paradigm allows the measurement of the putatively dissociable components of fear (FI, referred to as one-way avoidance) and anxiety (RAI, referred to as two-way avoidance). Behavioural data were acquired using a force-sensing joystick apparatus (PH-JS1, Psyal, London, UK). The harder the joystick was pushed, the faster the cursor (green dot) moved.

**Figure 2.**
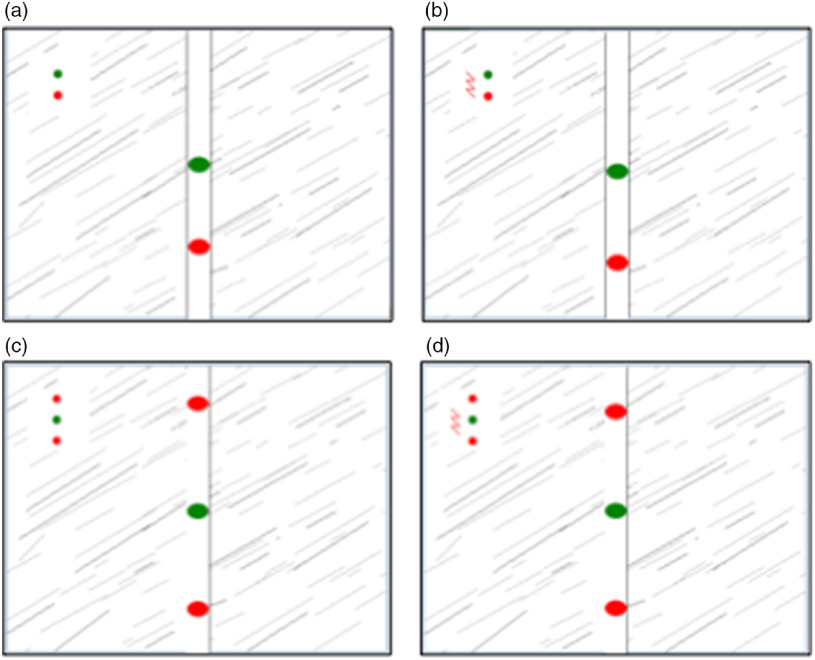
(a and b). One-way avoidance, labelled flight intensity (FI); (a) (on the left, without a lightning flash icon) A collision of the red and green dot led to no consequences; (b) (on the right, with a lightning flash icon) If the red dot collided with the green dot, participants received an unpleasant white burst. (c and d): Two-way avoidance, labelled risk assessment behaviour (RAI); (c) (on the left, without a lightning flash icon) A collision of one of the red dots and the green dot led to no consequences; (d) (on the right, with a lightning flash icon) If one of the two red dots collided with the green dot, participants received an unpleasant white burst.

As can be seen in Figure 2, a vertically orientated image of a runway was projected on the computer monitor. The green dot was representing the participant and movable by pushing the joystick. Contrary to our first study but in accordance to our second study from 2013, we did not calibrate the participants’ minimum force required for the green dot to reach escape velocity. Therefore, all participants had to spend the same strength to move the green dot, regardless of differences in absolute physical strength.

In addition to the green dot, one or two computer controlled red dots appeared on the screen. To control for individual differences in participants’ motor function and sedation effects of lorazepam, responses were measured with and without threat, as signalled by the presence or absence, respectively, of a lightning flash icon on screen. In the event of a collision, when the lightning flash icon was present, participants received an unpleasant but harmless 115-dB white noise burst of near instantaneous rise time lasting 250 ms through headphones.

In the one-way avoidance (Figure 2(a) and (b), representing FI), the cursor (green dot) was pursued by a single threat stimulus (red dot). The degree to which threat (presence of the lightning flash icon, Figure 2(b)) increased the velocity of the green dot along the runway is related to FI. Backward movements were not possible. FI scores were calculated as the average velocity in trials that contained no threat of white noise (Figure 2(a)) subtracted from the average velocity in trials with a threat of white noise (Figure 2(b)).

The two-way active avoidance tasks (Figure 2(c) and (d), representing RAI) were identical, except that a second red dot travelled ahead of the green dot at a constant velocity, causing a goal conflict whereby the participant had to travel fast enough to avoid the pursuing threat, but not so fast that they collided with the leading threat stimulus. The degree to which threats (as signalled by the lightning flash icon) increased the magnitude of forward–backward oscillation of the participants when trapped between the two red dots represents RAI-related goal conflicts. Similar to the calculation of FI scores, RAI scores were calculated as the standard deviation (SD) of the average velocity in trials that contained no threat of white noise (Figure 2(c)) subtracted from SD of the average velocity in trials with a threat of white noise (Figure 2(d)).

Each testing session consisted of 12 trials of each of the above described types. In total, 48 trials were presented in a pseudo-random order as well as the intertrial intervals were varied pseudo-randomly (between 15 and 30 s). Both experimental manipulations served to enhance unpredictability. Trials terminated automatically by collision between the green and the red dot. Therefore, in threatening trials, participants were only briefly exposed to white noise. Each trial had a maximum duration of 7 s. Trials without collision terminated automatically after this duration. An early collision did not alter the overall duration of the trial.

### 2.5 Saccade task

Saccades were recorded using an EyeLink 1000 video-based eye-tracker (SR Research Ltd., Ontario, Canada), which records at a sampling frequency of 1,000 Hz. Participants were seated 70 cm away from the centre of the computer screen (1600 × 1050 pixels, 60 Hz refresh rate) with their head resting on a chin rest to stabilize head position. A five-point horizontal/vertical calibration was conducted prior to the task.

At the beginning of each trial, a central fixation point, a white circle approximately 0.35° of visual angle in diameter, was presented on a black background in the middle of the computer screen. The central fixation point was presented for 500–1,500 ms and was followed by a peripheral target of identical dimensions and appearance presented in one of four locations (±7.25°; ±14.5° horizontally from the fixation point). Participants completed 60 trials in total, with each location presented 15 times, in random order. Participants were instructed to look at the fixation point as soon as it appeared on the screen and to redirect their gaze to the peripheral target when it appeared as fast and accurately as possible.

Saccades were identified using the Data Viewer software package (SR Research) and defined as eye movements having a minimum amplitude of 1°, a minimum velocity of 30°/s and a minimum latency to peripheral stimulus of 70 ms. The dependent variable was the peak velocity (in °/s) of the first valid saccade to the peripheral stimulus. Building on findings that sedation reliably leads to reduced saccadic peak velocity (de Visser et al., 2003; Ettinger et al., 2018; Reilly, Lencer, Bishop, Keedy & Sweeney, 2008), this measure was used as an objective behavioural marker of the sedative effects of lorazepam in the absence of exposure to threat.

### 2.6 Questionnaires

In order to replicate our earlier findings (Perkins et al., 2009, 2013), we chose the same questionnaires as in those studies. First, the FSS (Wolpe & Lang, 1974) was administered. The FSS consists of two major subscales. Social fear measures a mixture of trait anxiety and fear (Cooper et al., 2007). Tissue-damage fear constitutes a relatively pure measure of fear (Perkins et al., 2007). Second, we administered the STAI (Spielberger et al., 1983) as a specific measurement of anxiety. Contrary to our study in 2013, we did not administer the Eysenck Personality Questionnaire – Revised (Eysenck & Eysenck, 1991), as we found during that study that the EPQ neuroticism dimension correlates so strongly with trait anxiety (*r* = .825) that it is effectively redundant.

### 2.7 Statistics

Statistical analyses were performed using the Statistical Package for the Social Sciences (SPSS version 25). Reliabilities of personality measures were calculated in terms of internal consistencies (Cronbach’s α). Associations between dependent variables in the JORT and scores on FSS and STAI-T were assessed using Pearson’s product–moment correlations. For the analysis of effects of drug on the intensity of threat-avoidance behaviour, participants with scores of three SDs above or below mean scores of the sample were considered to be outliers and excluded. For this reason, 2 participants were excluded for the analysis of FI (final sample *N* = 48, 26 females) and 10 participants were excluded for the analysis of RAI (final sample *N* = 40, 21 females). Learning effects were calculated by repeated measures analysis of variance (ANOVA), with the three testing sessions as a three-level-within-subjects factor. To investigate the effects of lorazepam, further ANOVAs, with dosage (placebo, 0.5 and 1 mg lorazepam) as a three-level-within-subjects factor were calculated. In a further step, scores of the three personality questionnaires scales were divided at the sample’s median. Then, these dichotomous variables were entered as a between-subject factor into separate repeated measures ANOVAs. To control for gender effects, gender was entered as an additional between-subject factor into the repeated measures ANOVAs.

Finally, to control for sedative effects of lorazepam, we calculated a further repeated measures ANOVA with the three different drug conditions as within-subjects factor and prosaccade peak velocity as dependent variable. Five participants with less than 3 valid trials in one or more of the four locations were excluded from peak velocity analyses. Also, the prosaccade peak and the JORT variables FI and RAI were correlated in order to test if possible drug effects on fear and anxiety were related to general sedative effects of lorazepam. For all ANOVAs with significant results, post-hoc t-tests for dependent samples were calculated to identify which particular differences between pairs of means are significant. Moreover, post-hoc power analyses were calculated to assess the statistical power of all ANOVAs and significant results were Bonferroni corrected for multiple testing.

## 3. Results

### 3.1 Reliabilities of personality measures

The reliabilities in terms of internal consistencies (Cronbach’s α) were as follows: STAI-T: *n* = 20, α = .91, tissue-damage fear: *n* = 32, α = .90; social fear: *n* = 32, α = .93.

### 3.2 Personality effects on task performance

Descriptive statistics and intercorrelations of self-report and performance measures are depicted in Table 1. In terms of correlations with defensive behaviour, bivariate correlations only showed a significant correlation between FSS tissue-damage fear and RAI in the placebo condition. There were no further significant correlations between self-reported questionnaires scores with dependent behavioural variables in the JORT.

**Table 1.** Means, standard deviations and intercorrelations of self-reported and behavioural measures of defensive behaviour

Variable	Mean (SD)	1	2	3	4	5	6	7	8
1. Trait anxiety	33.8 (7.99)	–							
2. Tissue-damage fear	18.16 (11.77)	0.343*	–						
3. Social fear	24.34 (14.43)	0.575**	0.761**	–					
4. FI (placebo)	0.51 (0.79)	−0.185	−0.161	−0.246	–				
5. FI (0.5 mg lorazepam)	0.59 (0.76)	−0.077	−0.006	−0.020	0.277	–			
6. FI (1 mg lorazepam)	0.51 (0.64)	0.037	−0.249	−0.084	0.259	0.472**	–		
7. RAI (placebo)	0.11 (0.14)	0.020	0.331*	0.179	0.394*	0.364*	0.164	–	
8. RAI (0.5 mg lorazepam)	0.00 (0.25)	−0.118	−0.197	0.104	0.027	−0.064	−0.008	−0.151	–
9. RAI (1 mg lorazepam)	0.09 (0.17)	−0.126	0.091	0.159	0.056	0.178	0.495**	0.184	0.055

Correlations reflect Pearson’s product–moment correlation coefficients. *p < .05; **p < .01.

### 3.3 Learning effects on RAI and FI

Evaluating the behavioural JORT data according to the time-point of testing, there was a learning effect neither on RAI (*F*_(2,78)_ = 0.900, *p* = .411, power: .200) nor on FI (*F*_(1.576,74.053)_ = 0.062, *p* = .902, power: .058).

### 3.4 Effects of lorazepam on RAI and FI

**Figure 3.**
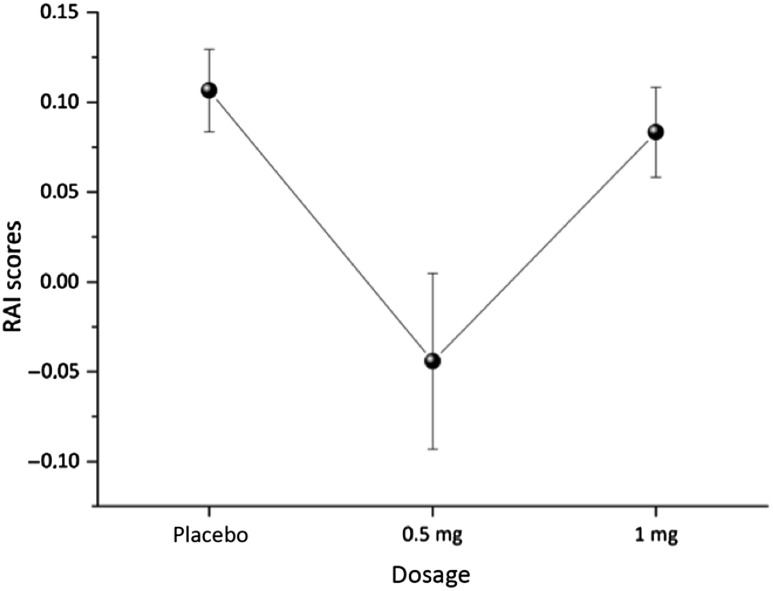
Main effect of dosage on RAI, means and standard error of means (SEM).

Results show a significant main effect of dosage on RAI (*F*_(2, 78)_ = 5.178, *p* = .008, η^2^ = .117, power = .814; see Figure 3). After Bonferroni correction for multiple testing, this effect is still significant. It also remained significant when outliers were included (*F*_(2,98)_ = 3.718, *p* = .028, η² = .071, power: .669).

Post-hoc tests showed significant lower RAI scores in the 0.5 mg condition than in the placebo and the 1 mg condition. There was no significant difference between the placebo and the 1 mg condition. Also, there was no main effect of dosage on FI (*F*_(2,94)_ = 0.346, *p* = .708, power: .104).

### 3.5 Interaction effects of lorazepam and personality on RAI

There was neither a main effect of STAI-T (*F*_(1,38)_ = 0.817, *p* = .372, power: .143) nor an interaction effect of STAI-T by dosage on RAI (*F*_(1.727,65.634)_ = 0.811, *p* = .433, power: .184). Regarding the FSS, similar results were observed: There was a main effect neither of FSS social fear on RAI (*F*_(1,38)_ = 3.154, *p* = .084, power: .410) nor of FSS tissue damage on RAI (*F*_(1,38)_ = 1.903, *p* = .176, power: .270). Also, there was neither an interaction effect of FSS social fear by dosage on RAI (*F*_(2,76)_ = 0.087, *p* = .917, power: .063) nor an interaction effect of FSS tissue damage by dosage on RAI (*F*_(2,76)_ = 1.102, *p* = .337, power: .237).

### 3.6 Interaction effects of lorazepam and personality on FI

Results regarding interaction effects of lorazepam and personality on FI are similar to the RAI results. There was neither a main effect of STAI-T (*F*_(1,46)_ = 0.033, *p* = .856, power: .054) nor an interaction effect of STAI-T by dosage on FI (*F*_(1.769,81.371)_ = 1.305, *p* = .275, power: .260). Also, interactions with the FSS questionnaire showed no significant results: There was neither a main effect of FSS social fear (*F*_(1,46)_ = 0.529, *p* = .471, power: .110) nor an interaction effect of FSS social fear by dosage on FI (*F*_(2,92)_ = 1.037, *p* = .359, power: .148), and there was neither a main effect of FSS tissue damage (*F*_(1,46)_ = 0.858, *p* = .359) nor an interaction effect of FSS tissue damage by dosage on FI (*F*_(2,92)_ = 1.441, *p* = .242, power: .301).

### 3.7 Gender effects

There was neither a main effect nor two-way interactions (gender by dosage, gender by personality) or a three-way interaction (gender by dosage by personality) effect of gender on RAI or FI. All interactions with personality were tested separately for the three variables FSS social fear, FSS tissue damage and STAI-T.

### 3.8 Drug effects on saccadic peak velocity

Repeated measures analysis of variance showed a significant main effect of drug condition on prosaccade peak velocity (F_(2,88)_ = 12.274, *p* < .001, η2 = .218, power: .995). Additional analyses showed that the 0.5 mg lorazepam condition significantly decreased peak velocity as compared to placebo (T_(44)_ = 2.878, *p* = .006; see descriptive statistics of the saccadic peak velocity in Table 2). One mg lorazepam significantly decreased the peak velocity compared to the placebo condition (T_(44)_ = 4.855, *p* < .001) as well as compared to the 0.5 mg lorazepam condition (T_(44)_ = 2.110, *p* = .041). Table 2 shows correlations between prosaccade peak velocity and the JORT variables of FI and RAI. No significant correlations between the lorazepam effects were observable, indicating that sedative effects of lorazepam were not responsible for altering the defensive behaviour of participants.

**Table 2.** Means, standard deviations and correlations of defensive behaviour and eye movements.

Variable	Mean (SD)	1	2	3
1. Prosaccade Peak Velocity placebo	337.59 (41.43)	*–*		
2. Prosaccade Peak Velocity 0.5 mg	324.89 (47.55)	.787**	*–*	
3. Prosaccade Peak Velocity 1 mg	315.40 (48.15)	.776*	.801**	–
4. Flight Intensity placebo	.550 (.76)	.112	.009	.047
5. Flight Intensity 0.5 mg	.562 (.72)	–.182	–.124	–.043
6. Flight Intensity 1 mg	.509 (.63)	–.044	–.076	–.208
7. Risk Assessment placebo	.110 (.15)	–.110	.019	.095
8. Risk Assessment 0.5 mg	–.000 (.21)	–.055	.038	.076
9. Risk Assessment 1 mg	.088 (.16)	.003	.079	–.150

Correlations reflect Pearson’s product–moment correlation coefficients *P<0.05; **P<0.01.

## 4. Discussion

Building on the results of two previous studies of our group, which demonstrated effects of lorazepam on defensive behaviour, the aim of the present study was to partially replicate the findings and to extend our knowledge on lower dosage threshold and moderating effects of personality. Given that the results of the studies by Perkins et al. (2009, 2013) showed some inconsistencies, the need for a replication study was apparent. The discrepancies between the studies were particularly evident with regard to the dosage of lorazepam showing effects on defensive behaviour. As putative causes, we assumed differences in sample size and gender distribution across studies. Therefore, we increased the sample size and aimed for an approximately equal gender distribution. Also, we introduced a lower dosage of lorazepam (0.5 mg) to obtain information on lorazepam’s threshold effects. Apart from this, the present study protocol was very similar to the one used in Perkins et al. (2013).

Although the present study showed an effect of lorazepam on defensive behaviour independent of sedative effects of the drug as assessed by changes in peak velocities in the prosaccades, the discrepancies between the two earlier studies could not be conclusively clarified. In terms of main effects, in the Perkins et al. (2009) study there was a significant main effect of drug on RAI but not on FI. In the Perkins et al. (2013) study, there was no main effect of drug on RAI but on FI. In terms of personality modulation in Perkins et al. (2009), RAI was reduced by 1 mg of lorazepam in participants scoring low on FSS social fear. This was not observable in participants with high FSS social fear scores. The results in Perkins et al. (2013) presented a different picture. A 2 mg dose of lorazepam reduced RAI but only in participants scoring low in STAI-T. In the FI condition, lorazepam caused a dose-dependent increase on FI in participants scoring low on FSS tissue-damage fear and a dose-dependent decrease on FI in high-scoring participants on FSS tissue-damage fear. In the present study, none of these results were replicated except the main effect of lorazepam on RAI, but this was driven by the 0.5 mg dose of lorazepam – the 1 mg dose did not separate significantly from placebo.

**Figure 4.**
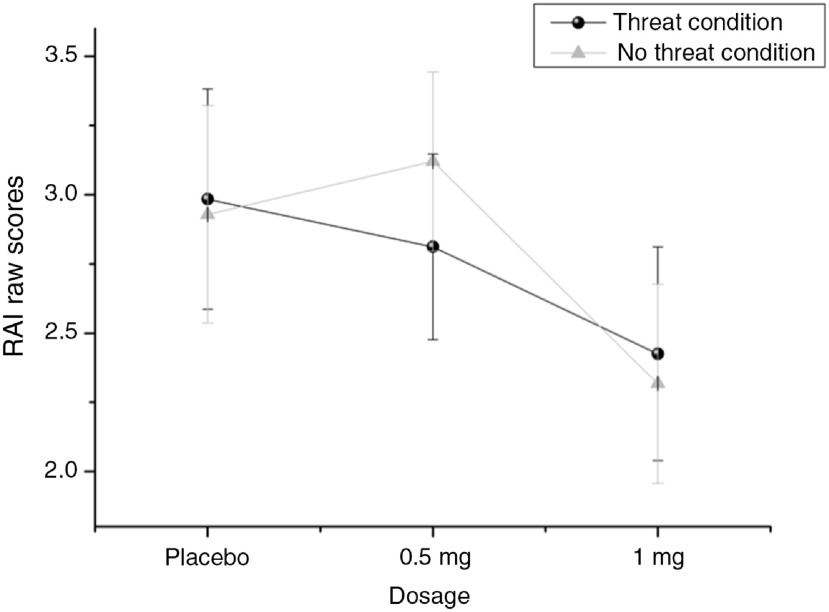
Results of supplementary post-hoc analyses with respect to RAI raw scores.

To explain the unexpected effect of no difference between the 1 mg dose and placebo, we critically analysed the computation of our RAI score in the JORT paradigm. As described in the method section, the dependent variable is calculated as the SD of the average velocity in trials that contained no threat of white noise (Figure 2(c)) subtracted from the SD of the average velocity in trials with a threat of white noise (Figure 2(d)). Thus, the RAI score represents a difference between two variables and, consequently, the dependent variable can be influenced by two different parameters. First, RAI scores decrease when the SD of the average velocity in trials with a threat of white noise decreases and the SD of the average velocity in trials with no threat is unchanged. This is what we expect as an actual anxiolytic effect of lorazepam. Second, it could also be that a reduced RAI score is due to a higher velocity in trials that contained no threat of white noise than in trials with a threat. In order to test both possibilities, we looked at the velocities in the threat and the no threat conditions separately for each dosage (placebo, 0.5 and 1 mg). The pattern of results for this additional analysis can be seen in Figure 4.

It can be seen that the effect of 0.5 mg lorazepam is driven by both factors: as expected, the SD of the average velocity in trials that contained a threat of white noise decreased, but additionally, the SD in trials that contained no threat of white noise increased, thereby strengthening the decrease of the RAI score representing the difference between both conditions. Of particular interest is the pattern of results in the 1 mg condition as compared to the placebo condition because the RAI scores (difference scores) in these two conditions do not differ but the absolute values in the threat and the no threat condition differ strongly (higher values in the placebo condition than in the 1 mg condition). Overall, on the descriptive level, there is an anxiolytic effect of lorazepam irrespective of dosage if only the raw data in the threat condition are considered (higher scores in the placebo condition than in both lorazepam conditions). Therefore, the significant effect in the 0.5 mg condition is caused not only by the anxiolytic effect in the threat condition but also by the relatively high scores in the no threat condition. In sum, the additional analysis suggests that the trials without threat are also aversive and therefore are able to evoke risk assessment and accordingly are sensitive to the influence of lorazepam. From these results, we derive that a low dosage of 0.5 mg is particularly effective in high threatening situations but less in low threatening situations. But by increasing the dosage, also lower threatening situations are influenced by the anxiolytic effects of lorazepam. The question of where the exact threshold between high and low threatening situations remains open and needs further research. The relatively high RAI scores in the no threat condition can be explained by the nature of the JORT paradigm (Perkins et al., 2019). The entire JORT situation is an anxiety-inducing approach to threat and goal conflict in that participants are volunteering to enter a testing room where they know (due to the informed consent process) they will receive a nasty burst of white noise. So this idea does make it likely there will be some anxiety experienced across the entire task, even in the non-threat RAI trials a hypothesis that is supported by the additional findings depicted in Figure 4. Perkins et al. (2019) described this phenomenon “the experiment knowledge problem”, something which does not affect rodents because they do not realize they are in an experiment.

Taking into account our new data, the overall view of the drug effects in all three studies suggests that lorazepam does alter defensive behaviour in humans but that the patterning of that effect is highly variable and depends on certain conditions as described above. It is unlikely that the patterns of results are due to gender effects. In Perkins et al. (2009), only male participants were included, but neither in Perkins et al. (2013) nor in the current study, interactions between drug effects and gender were observed. Furthermore, we cannot explain our divergent results by our larger sample size, because due to the exclusion of 10 participants in the two-way avoidance condition, the sample was nearly of the same size compared to Perkins et al. (2013).

We found a significant main effect of drug condition on FI in Perkins et al. (2013) study but not in Perkins et al. (2009) or the present study. Furthermore, in Perkins et al. (2009) and in the current study, there were no interaction effects between fear and anxiety questionnaires and dosage of lorazepam on FI. This was different in Perkins et al. (2013): There was a significant interaction between dosage of lorazepam and scores on FSS tissue-damage fear. In participants scoring high on FSS tissue damage, FI scores were decreased, whereas in participants scoring low on FSS tissue damage FI scores were increased. Post-hoc contrasts showed that this interaction was due to the difference between the 2 mg condition and the placebo condition. Unfortunately, we are not able to test this interaction in the Perkins et al. (2009) or in the current study, as 2 mg lorazepam was not administered in those studies.

Some limitations of the present study have to be noted. First, there are shortcomings regarding the operationalization of defensive behaviour inherent to the JORT paradigm. JORT is not able to measure all components of defensive behaviour. On the one hand, this concerns the measurement of anxiety. For example, anxiety-related hyper-scanning or checking of memory traces could not be operationalized. Approach withdrawal oscillation constitutes an important facet of anxiety, but the other components of anxiety are also of importance. On the other hand, Gray (Gray & McNaughton, 2000) postulated three different components of fear behaviour, namely fight, flight and freezing. In the JORT, only flight behaviour is operationalized. Participants can only push the joystick forward in order to escape; not pushing the joystick (freezing) is not an alternative, as than the collision is inevitable. Alternative behavioural options reflecting fight behaviour are not included in the paradigm at all.

Another point concerns the knowledge available about the experimental situation including particularly the information on being threatened by a very unpleasant noise (see above). This is in contrast to the animal studies where rodents were struck by the threat unexpectedly (Blanchard & Blanchard, 1989). Therefore, the definition of fear-related behaviour, as solely threat avoidant, is not reflected in the JORT. The rationale is that the entire testing session may be understood as an approach-to-threat situation which should theoretically elicit anxiety. This assumption complicates a clear differentiation of fear and anxiety within the JORT paradigm and could be related to the heterogeneous findings in our three studies. In particular, FI scores are more difficult to interpret, also considering the influence of lorazepam and the interactions with personality questionnaires. For ethical reasons, it is difficult to modify the experimental conditions in a way that the threat hits the participants by surprise without being informed prior to testing. However, the operationalization of FI was consistent across all three studies and, therefore, it can be excluded that this is the explanation for the inconsistent findings.

Interindividual differences in the perception of task difficulty can be a further limitation of the JORT paradigm. The requirements, which must be met to prevent a collision of the green dot with any red dot, could be easier for some participants than for others. It may also be possible that the requirements are too easy for individual participants and therefore neither anxiety nor fear is triggered in these participants. Also, the familiarity with similar games that are either also played with a joystick or games with a similar design (e.g. the arcade game Pac-Man) can matter. So far, we did not include questions covering these possible influences on performance in the JORT paradigm.

Finally, it can be argued that the JORT lacks ecological validity. At least for some participants it may seem more like a game which has little to do with the fears and threats of daily life, especially complex, abstract social anxiety which is likely to be more salient to humans than rodents. Creating an experimental situation with more ego-involvement of the participants, that shows more resemblance to reality and that is also able to differentiate all three sub-components of the FFFS, would be favourable.

## 5. Conclusions

Overall, and in line with Gray’s r-RST, the present study demonstrates anxiolytic effects of lorazepam in dosages ≤1 mg in the absence of a drug effect on fear using a translational behavioural task. However, the moderating role of personality on defensive behaviour has to be clarified in future research.

## Supplementary material

For supplementary material accompanying this paper visit https://doi.org/10.1017/pen.2020.1. click here to view supplementary material
