# Supplementary material for: Differentiating anxiety from fear: an experimental–pharmacological approach
Source: Personal Neurosci. 2020 Jun 17;3:e6. doi: 10.1017/pen.2020.1 (PMC7303801; doi:10.1017/pen.2020.1)
Supplement: Supplementary file 1 [file S2513988620000012sup.zip › S2513988620000012sup001.docx]

**Supplementary Material**

Tab. S1: Overview on the results of previous and current JORT-studies

| *Study* | *Perkins et al., 2009* | *Perkins et al., 2013* | *Lippold et al., 20xx* |
| --- | --- | --- | --- |
| sample | 30 males | 20 males  20 females | 27 females  23 males |
| drug and dosage | - Placebo - 10 mg citalopram - 1 mg lorazepam | - Placebo - 1 mg lorazepam - 2 mg lorazepam | - Placebo - 0.5 mg lorazepam - 1 mg lorazepam |
| questionnaires | - *Fear Survey Schedule* (FSS; Wolpe and Lang, 1977) - *Trait Scale; Spielberger State-Trait Anxiety Inventory* (STAI-T, Spielberger et al., 1983) | - FSS - STAI-T - Eysenck Personality Questionnaire – Revised (Eysenck & Eysenck, 1991) | - FSS - STAI-T |
| main results | - lorazepam modulated RAI: lorazepam reduced RAI in participants scoring in the lower half of the sample on FSS social fear - no effect of lorazepam on FI - citalopram neither showed an effect on RAI nor on FI | - RAI was affected by lorazepam but the effect was modulated by personality - 2 mg lorazepam reduced RAI in low scorers on trait anxiety and increased RAI in high scorers - personality had no differential effects in the placebo and 1 mg lorazepam conditions - Lorazepam increased FI in the participants with low scores on FSS tissue damage in a dose-dependent manner, whereas FI was decreased in participants with high FSS tissue damage scores in a dose-dependent manner | - main effect of 0.5 mg lorazepam on RAI - no significant difference between 1 mg lorazepam and placebo |

**
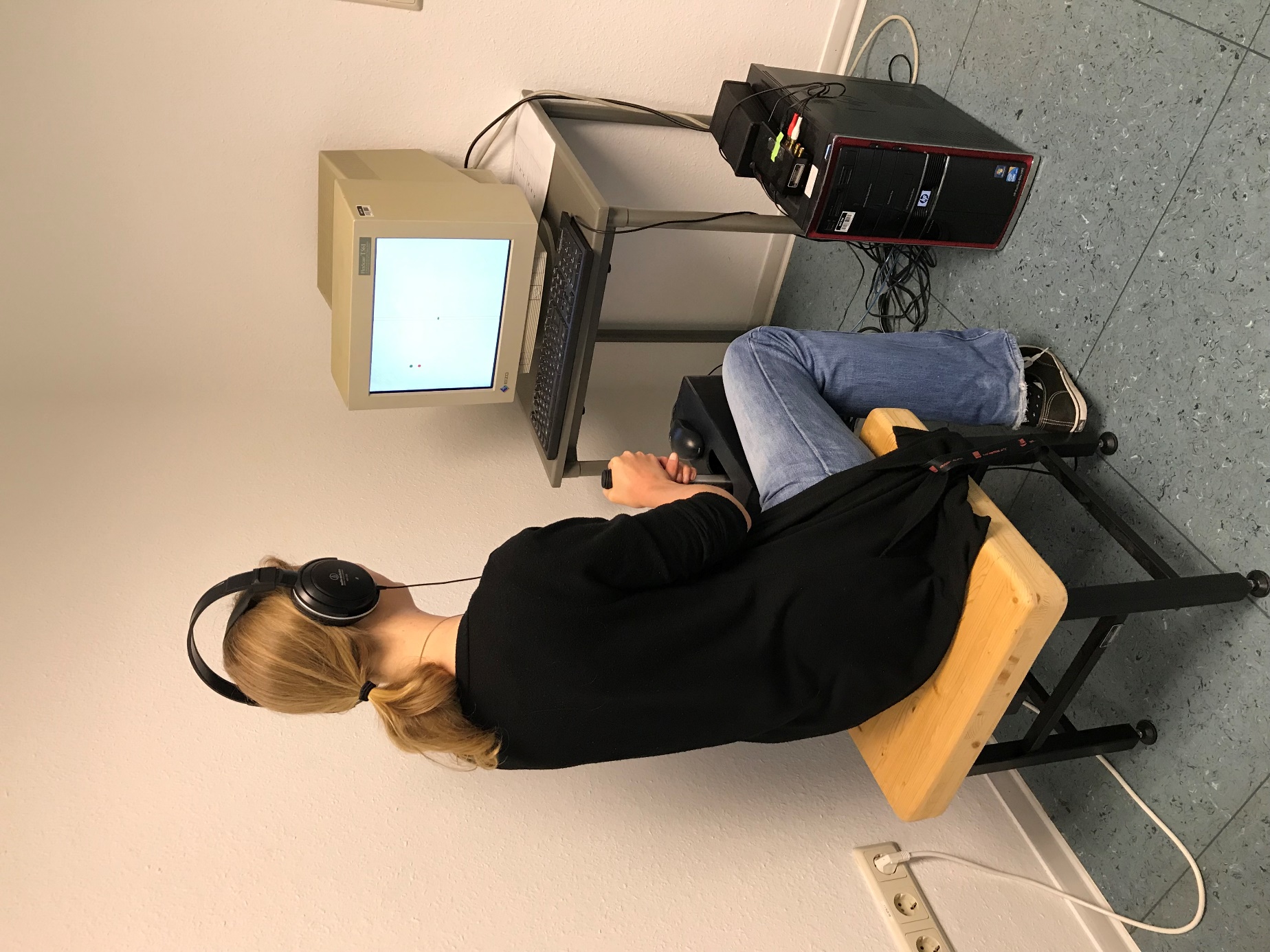
**

Fig. S1: JORT-apparatus in the laboratory
